# PTTH–Torso Signaling System Controls Developmental Timing, Body Size, and Reproduction through Regulating Ecdysone Homeostasis in the Brown Planthopper, *Nilaparvata lugens*

**DOI:** 10.3390/ijms25105138

**Published:** 2024-05-09

**Authors:** Xumei Luo, Jinli Zhang, Chuanxi Zhang, Naiming Zhou

**Affiliations:** 1 Institute of Biochemistry, Zhejiang University, Hangzhou 310058, China; luoxumei@zju.edu.cn; 2 Institute of Insect Science, Zhejiang University, Hangzhou 310058, China; zhangjinli@caas.cn; 3 State Key Laboratory for Managing Biotic and Chemical Threats to the Quality and Safety of Agro-Products, Ningbo University, Ningbo 315211, China

**Keywords:** prothoracicotropic hormone, Torso, *N. lugens*, 20-hydroxyecdysone

## Abstract

In holometabolous insects, such as *Drosophila* and *Bombyx*, prothoracicotropic hormone (PTTH) is well established to be critical in controlling developmental transitions and metamorphosis by stimulating the biosynthesis of ecdysone in the prothoracic glands (PGs). However, the physiological role of PTTH and the receptor Torso in hemimetabolous insects remains largely unexplored. In this study, homozygous PTTH- and Torso-null mutants of the brown planthopper (BPH), *Nilaparvata lugens*, were successfully generated by employing clustered regularly interspaced short palindromic repeats/CRISPR-associated 9 (CRISPR–Cas9). Further characterization showed that both *NlPTTH^−^^/−^* and *NlTorso^−^^/−^* mutants exhibited prolonged nymphal duration and increased final adult size. Enzyme-linked immunosorbent assay (ELISA) revealed that *NlPTTH^−^^/−^* and *NlTorso^−^^/−^* mutants exhibited a significant reduction in 20-hydroxyecdysone (20E) in fifth-instar nymphs at 48 h post-ecdysis compared to *Wt* controls. Furthermore, our results indicated that both *NlPTTH^−^^/−^* and *NlTorso^−^^/−^* mutants had shortened lifespan, reduced female fecundity, and reduced egg hatching rates in adults. These findings suggest a conserved role for the PTTH–Torso signaling system in the regulation of developmental transitions by stimulating ecdysone biosynthesis in hemimetabolous insects.

## 1. Introduction

In holometabolous insects, the complex physiological processes of molting and metamorphosis are initiated by a neuropeptide known as prothoracicotropic hormone (PTTH) [1,2,3,4,5]. PTTH was originally purified as a potential stimulant of the production of ecdysone in the prothoracic glands (PGs) from brain extracts of the silkworm *Bombyx mori* [6]. In lepidopteran insects, PTTH is biosynthesized within a bilateral pair of neurosecretory cells located in the brain, and subsequently conveyed to specialized neurohemal varicosities located on the corpus allatum (CA), an endocrine organ [7,8,9]. After release from CA, PTTH enters the hemolymph and binds to its receptor Torso (a receptor tyrosine kinase) located on the surface of prothoracic gland cells. This interaction activates the MAPK–ERK (mitogen-activated protein kinase–extracellular signal-regulated kinase) signaling cascade and ultimately leads to the biosynthesis and subsequent release of ecdysone (E) [10,11,12]. In *Drosophila melanogaster*, however, PTTH neurons directly innervate the PGs instead of the CA to regulate and control E synthesis via Torso–ERK signaling [2,13,14]. E, produced within the PGs, undergoes secretion into the hemolymph and subsequent conversion to its active form, 20-hydroxyecdysone (20E). This conversion process is facilitated by a 20-hydroxylase enzyme, denoted CYP314, predominantly localized within peripheral tissues such as the fat bodies and integument [15,16], which serves as the major molting hormone, binding to a nuclear receptor to induce diverse gene expression cascades and orchestrating the array of physiological, morphological, and behavioral transformations that characterize molting and metamorphosis [17].

The physiological significance of the PTTH–Torso pathway has already been extensively studied in holometabolous insects such as *D. melanogaster*, *Manduca sexta* and *B. mori* [2,18,19]. In *Drosophila*, genetic elimination of prothoracic gland (PG) neurons resulted in a significant delay in the onset of metamorphosis. *PTTH*-deficient larvae eventually underwent pupation and eclosion, and exhibited increased body size due to the prolonged larval feeding [2]. Similar to the absence of PTTH-producing neurons, targeted inhibition of Torso expression within the PGs results in a delay in pupariation initiation and a consequent increase in final pupal size, which is attributed to the extension of the larval growth period [14]. In *Bombyx*, knockout of *PTTH* resulted in developmental delays observed throughout both the larval and pupal stages. Some individuals experienced developmental arrest at the L2 stage, while others underwent early metamorphosis and pupation by the end of the fourth instar. *BmTorso* mutants also showed delayed development in both larval and pupal phases; however, all of the mutants progressed through five larval stages without encountering developmental arrest or experiencing premature metamorphosis [19,20].

Although PTTH–Torso signaling has been well characterized in *Drosophila* and lepidopteran insects and several PTTH homologues have been identified in some hemipteran insects [21,22], few studies have directly addressed the function of PTTH and Torso in hemipteran insects, except for in vitro RNAi-mediated knockdown of PTTH in *Rhodnius prolixus*, which resulted in reduced ecdysteroid production [22]. The brown planthopper (BPH), scientifically known as *N. lugens* (Hemiptera: Delphacidae), represents a significant hemimetabolous pest of rice, causing considerable damage to rice crops by extracting phloem sap from the rice plant [23]. Here, we generated stable homozygous *PTTH* (*NlPTTH*) and *Torso* (*NlTorso*) mutants through the utilization of the CRISPR–Cas9 system. *NlPTTH^−^^/−^* and *NlTorso^−^^/−^* mutants exhibited prolonged nymphal duration and increased body, as evidenced by significantly reduced 20E titers in fifth-instar nymphs at 48 h post-ecdysis compared to *Wt* controls. In addition, *NlPTTH^−^^/−^* and *NlTorso^−^^/−^* mutants showed defects in lifespan, fecundity and hatching rate. Our results suggest that PTTH–Torso signaling plays a crucial role in the nymphal development of BPH and influences normal adult body size and physiology. The PTTH–Torso signaling system serves as a conserved mechanism governing developmental transitions across both holometabolous and hemimetabolous insects.

## 2. Results

### 2.1. Identification and Sequence Analysis of NlPTTH and NlTorso

**Figure 1:**
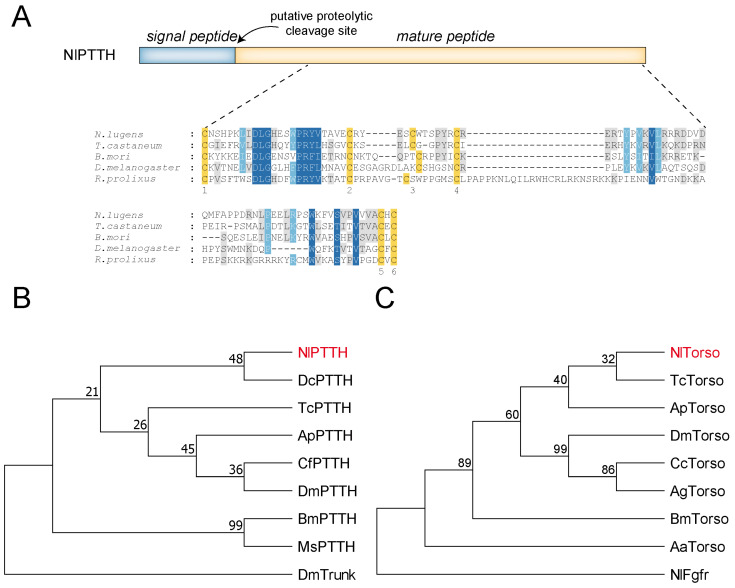
Sequence analyses of NlPTTH and NlTorso proteins. (**A**) Schematic of NlPTTH domains and sequence alignment. It is hypothesized that processing of the precursors leads to the release of mature C-terminal peptides, which subsequently assemble into cysteine knot-type structures. Cysteines are highlighted in yellow and numbered 1 to 6, conserved residues are blue, conservative substitutions are light blue, and low similar residues are gray. (**B**,**C**) Phylogenetic analysis of PTTH and Torso orthologues across various species was conducted utilizing amino acid sequences. *Nl*, *N. lugens*; *Tc*, *Tribolium castaneum*; *Bm*, *B. mori*; *Dm*, *D. melanogaster*; *Rp*, *R. prolixus*; *Ms*, *M. sexta*; *Dc*, *Diaphorina citri*; *Ap*, *Acyrthosiphon pisum*; *Cf*, *Camponotus floridanus*; *Cc*, *Ceratitis capitata*; *Ag*, *Anopheles gambiae*; *Aa*, *Aedes aegypti*. The phylogenetic tree, constructed with 1000 bootstrap replicates, was generated using the maximum-likelihood method in MEGA 11.

We identified *NlPTTH* and *NlTorso* by conducting searches within the genomic and transcriptomic data of BPH in our research group, employing existing insect gene sequences as reference queries. The CDS of *NlPTTH* was 387 bp long and exhibited complete sequence identity with the assembly derived from the genome sequencing (GenBank accession number XP_022196973.2), encoding 128 amino acid residues. The CDS of *NlTorso* was 2580 bp long and exhibited complete sequence identity with the assembly derived from the genome sequencing (GenBank accession number XP_039276982.1), encoding 859 amino acid residues. Protein sequence alignment of NlPTTH with PTTH homologues from three other insect species representing four orders (Coleoptera, Lepidoptera, Diptera, and Hemiptera) revealed shared conserved structures within the C-terminal region that constitute the mature peptide. Notably, all six cysteines crucial for intramonomeric bonds within the PTTH homodimeric molecule were preserved (Figure 1A) [24]. Phylogenetic analysis based on eight species showed that NlPTTH and NlTorso have a close genetic relationship with relative counterparts from other insects, thus confirming the authenticity of the identified sequences (Figure 1B). DmTrunk (*D. melanogaster* Trunk) and NlFgfr (*N. lugens* fibroblast growth factor receptor homologue) served as the outgroup control, which was separated from the PTTH and Torso branches.

### 2.2. Spatiotemporal Expression and In Situ Hybridization Chain Reaction (HCR)

**Figure 2:**
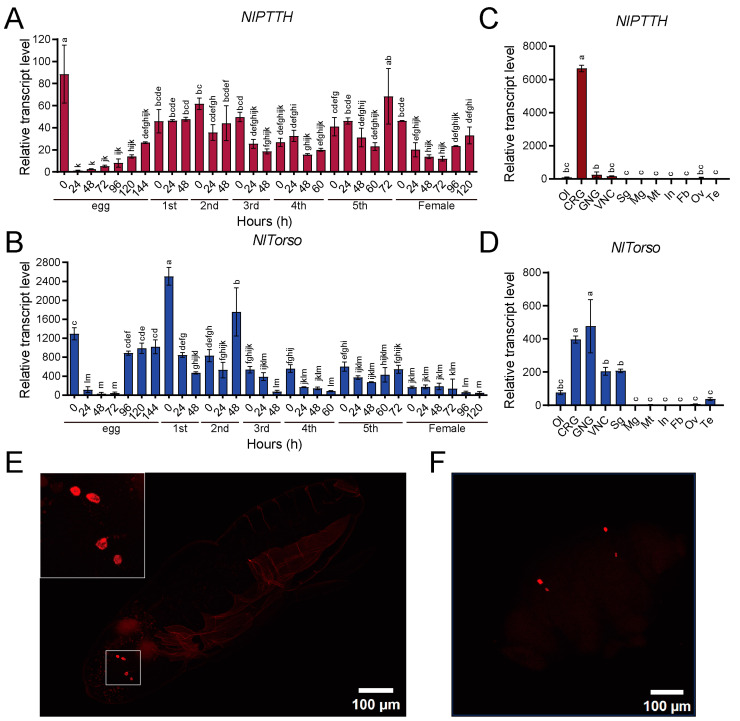
Spatiotemporal expression and in situ HCR. (**A**,**B**) Temporal expression patterns of *NlPTTH* and *NlTorso* throughout all developmental stages. (**C**,**D**) Tissue-specific expression profiles of *NlPTTH* and *NlTorso* in optic lobes (Ol), central brain (CR), gnathal ganglia (GNG), ventral nerve cord (VNC), salivary glands (Sg), midgut (Mg), Malpighian tubules (Mt), integument (In), fat body (Fb), ovaries (Ov) and testis (Te). (**E**) Expression of *NlPTTH* in 96 h BPH embryos. (**F**) Expression of *NlPTTH* in the cerebral ganglia (CRG) of fifth-instar BPH nymphs. Statistical analyses in (**A**–**D**) were performed using one-way ANOVA. Different lowercase letters mean significant differences (*p* < 0.05).

We investigated the spatiotemporal expression of *NlPTTH* and *NlTorso* using qRT-PCR. At the embryonic stage, *NlPTTH* and *NlTorso* had significant transcript levels in newly laid and late-developing eggs, and were almost undetectable at 24, 48, and 72 h. *NlPTTH* and *NlTorso* were consistently detected in both nymphal and adult stages, with peak expression occurring around each molt (Figure 2A,B). Tissue-specific analysis showed that *NlPTTH* expression levels were significantly higher in the central brain (CR) (Figure 2C). *NlTorso* transcripts were detected at relatively high levels in the CR and the gnathal ganglia, at relatively low levels in the ventral nerve cord and the salivary glands, and nearly undetectable in the optic lobes and the testes (Figure 2D).

To confirm the distribution of *NlPTTH*, we conducted in situ HCR in 96 h embryos and dissected BPH brains. *NlPTTH* was detected in two pairs of neurosecretory cells located in the dorsolateral region of the protocerebrum (Figure 2E).

### 2.3. Establishment of Homozygous NlPTTH and NlTorso Mutant Lines

**Figure 3:**
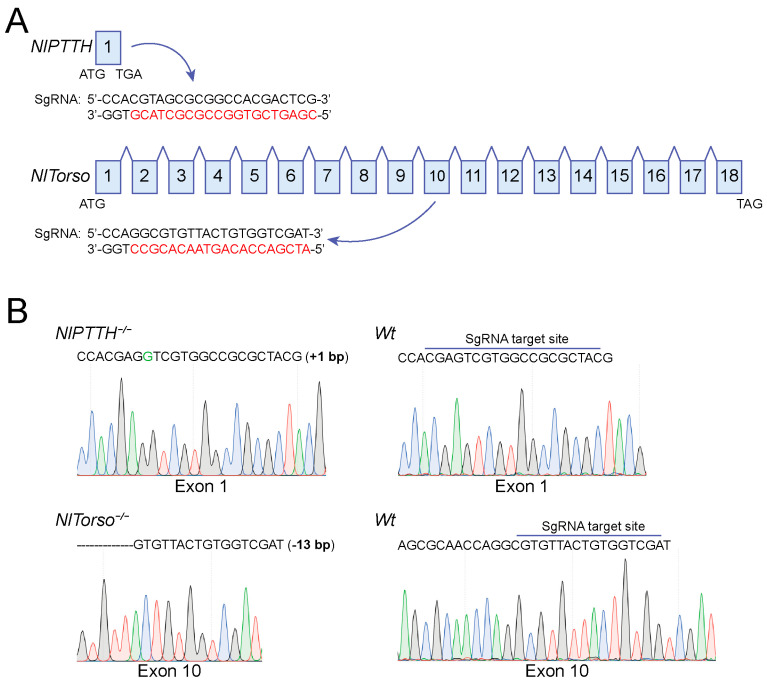
Establishment of homozygous *NlPTTH* (*NlPTTH^−/−^*) and *NlTorso* (*NlTorso^−/−^*) mutant lines. (**A**) Schematic representation of the specific sgRNA target sites within exon 1 and exon 10 of *NlPTTH* and *NlTorso*, respectively. The exons encompassing the CDS of *NlPTTH* and *NlTorso* are indicated by numerical values. The target sequences used to generate mutations in *NlPTTH* and *NlTorso* are highlighted in red. (**B**) Sanger sequencing to confirm *NlPTTH^−/−^* and *NlTorso^−/−^* mutants. The genomic DNA covering the target sites of *Wt*, *NlPTTH^−/−^* and *NlTorso^−/−^* mutants were subjected to PCR amplification followed by Sanger sequencing. The sequence chromatograms show a 1 bp insertion (indicated by dashes) in exon 1 and a 10 bp deletion (also indicated by dashes) in exon 10 in *NlPTTH^−/−^* and *NlTorso^−/−^* mutants, separately compared to *Wt* controls. Color waves represent base calls.

To generate homozygous *NlPTTH* and *NlTorso* mutants, the intron–exon structures of both genes were determined through alignment of cDNAs with the BPH genomic sequence. *NlPTTH* and *NlTorso* CDS consisted of one and eighteen exons, respectively. We designed sgRNA targeting sites in exon 1 and exon 10 of the *NlPTTH* and *NlTorso* genes, respectively, for CRISPR–Cas9-mediated mutagenesis (Figure 3A). We microinjected a mixture of Cas9 mRNA and sgRNA into preblastoderm *Wt* eggs and reared them to adulthood (G0). G0 individuals were crossed with *Wt* adults to generate G1 offspring. Heterozygous mutants carrying a 1 bp insertion and a 13 bp deletion in *NlPTTH^+/−^* and *NlTorso^+/−^*, respectively, were isolated individually to obtain homozygous mutants. Heterozygous mutants were further backcrossed with *Wt* individuals, followed by inbreeding. The genotypes of the *NlPTTH^−/−^* and *NlTorso^−/−^* mutants were confirmed by PCR amplification using primers encompassing the target sites, and then sequenced by the Sanger method. Sequencing revealed the successful generation of homozygous *NlPTTH* and *NlTorso* mutants with a 1 bp insertion in exon 1 and a 13 bp deletion in exon 10, respectively, which were expected to completely abolish NlPTTH and NlTorso protein function. (Figure 3B).

### 2.4. NlPTTH^−/−^ and NlTorso^−/−^ Mutants Exhibit Prolonged Nymphal Duration and Increased Body Size

**Figure 4:**
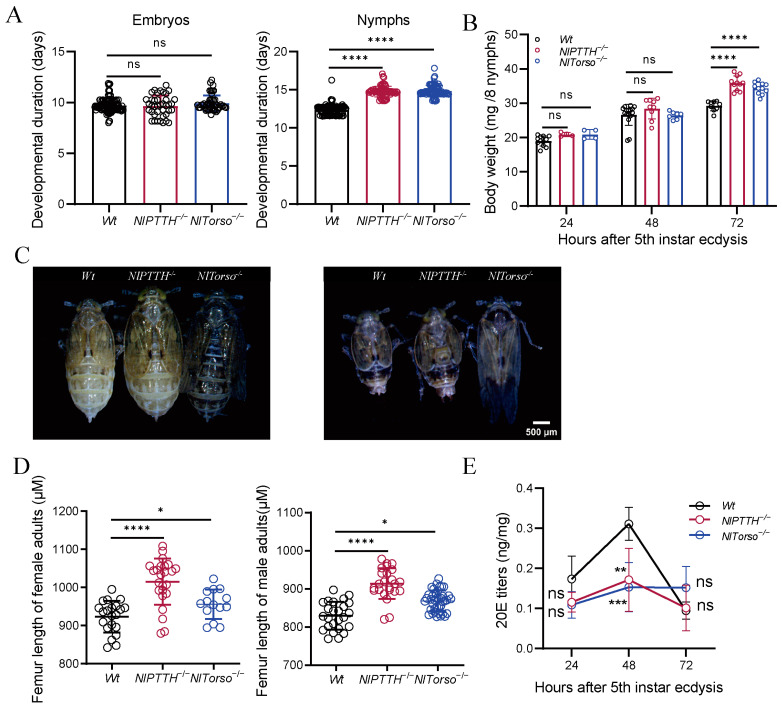
*NlPTTH^−/−^* and *NlTorso^−/−^* mutants exhibit prolonged nymphal duration and increased body size. (**A**) Duration of embryos and nymphs across *Wt*, *NlPTTH^−/−^* and *NlTorso^−/−^* mutants. (**B**) Body weight across *Wt*, *NlPTTH^−/−^* and *NlTorso^−/−^* mutants. (**C**) Body size of both female and male adults across *Wt*, *NlPTTH^−/−^* and *NlTorso^−/−^* mutants. (**D**) Femur length of both female and male adults across *Wt*, *NlPTTH^−/−^* and *NlTorso^−/−^* mutants. (**E**) 20E titers were measured in fifth-instar nymphs at 24, 48, and 72 h post-ecdysis for *Wt*, *NlPTTH^−/−^* and *NlTorso^−/−^* mutants. Statistical analyses in (**A**,**D**) were performed using two-tailed Student’s *t*-test (ns indicates no significant difference between two groups; * indicates significant difference between two groups at *p* < 0.05; **** indicates significant difference between two groups at *p* < 0.0001). Statistical analysis in (**B**,**E**) were performed using two-way ANOVA (ns indicates no significant difference between two groups; ** indicates significant difference between two groups at *p* < 0.005; *** indicates significant difference between two groups at *p* < 0.001; **** indicates significant difference between two groups at *p* < 0.0001).

To gain insights into the possible functions of *NlPTTH* and *NlTorso*, we aimed to investigate the effects of their absence. We observed that *NlPTTH^−/−^* and *NlTorso^−/−^* mutants had significantly prolonged nymphal durations of 14.77 and 14.84 days, respectively, compared to 12.37 days in *Wt*, but embryonic time was comparable to *Wt* controls (Figure 4A). Furthermore, we examined the body weight of fifth-instar nymphs at 24, 48, and 72 h post-ecdysis, and the findings revealed that the body weight of *NlPTTH^−/−^* and *NlTorso^−/−^* mutants at the 72 h mark of the fifth-instar nymph stage was significantly greater than *Wt* controls (Figure 4B). The prolonged nymphal duration of *NlPTTH^−/−^* and *NlTorso^−/−^* nymphs resulted in increased body size of both female and male adults compared to *Wt* controls (Figure 4C). To confirm this, we examined the femur lengths of both female and male adults, and the results showed that both *NlPTTH^−/−^* and *NlTorso^−/−^* adults had significantly longer femur lengths than *Wt* controls (Figure 4D).

To clarify whether the increased body size of *NlPTTH^−/−^* and *NlTorso^−/−^* mutants was caused by abnormalities in 20E titers, we examined 20E levels in fifth-instar nymphs at 24, 48, and 72 h post-ecdysis by enzyme-linked immunosorbent assay (ELISA). The results showed that 20E titers in fifth-instar nymphs at 48 h post-ecdysis of *NlPTTH^−/−^* and *NlTorso^−/−^* mutants were significantly reduced compared to *Wt* controls (Figure 4E).

### 2.5. Depletion of NlPTTH and NlTorso Impairs Adult Physiology in BPH

**Figure 5:**
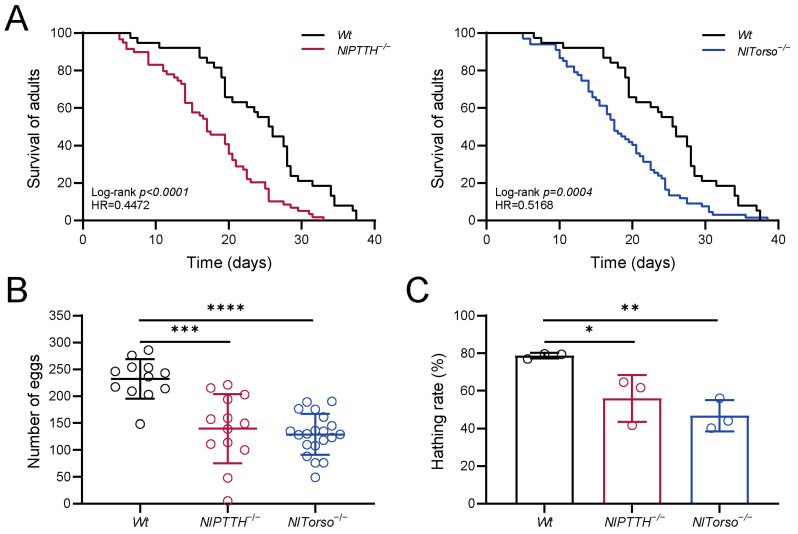
Depletion of *NlPTTH* and *NlTorso* impairs adult physiology in BPH. (**A**) Longevity of *Wt*, *NlPTTH^−/−^* and *NlTorso^−/−^* mutants. (**B**) Numbers of eggs deposited by *Wt*, *NlPTTH^−/−^* and *NlTorso^−/−^* mutants over a period of 10 days. (**C**) Hatching rate of eggs deposited by *Wt*, *NlPTTH^−/−^* and *NlTorso^−/−^* mutants. Statistical analysis in (**A**) was performed with the log-rank Mantel–Cox test. Statistical analyses in (**B**,**C**) employed two-tailed Student’s *t*-test (* indicates significant difference between two groups at *p* < 0.05; ** indicates significant difference between two groups at *p* < 0.005; *** indicates significant difference between two groups at *p* < 0.001; **** indicates significant difference between two groups at *p* < 0.0001).

To further investigate the effects of *NlPTTH* and *NlTorso* on the life history characteristics of BPH, we examined the lifespan, fecundity, and hatching rate of *NlPTTH^−/−^* and *NlTorso^−/−^* mutants. Compared to *Wt* controls, the median lifespan of *NlPTTH^−/−^* and *NlTorso^−/−^* adults decreased by 33.98% and 32.04%, respectively (Figure 5A). Furthermore, female *NlPTTH^−/−^* and *NlTorso^−/−^* individuals laid 39.85% and 44.46% fewer eggs, respectively, compared to *Wt* controls (Figure 5B). In addition, when compared to the robust hatching rate (78.69%) of *Wt* eggs, the hatching rate of *NlPTTH^−/−^* and *NlTorso^−/−^* eggs declined to 55.94% and 46.71%, respectively (Figure 5C). These results suggest that *NlPTTH* and *NlTorso* are required to optimize these life history traits in BPH.

## 3. Discussion

Ecdysteroids constitute a class of steroid hormones that precisely orchestrate numerous pivotal developmental events, such as molting and metamorphic transitions in arthropods [25]. In holometabolous insects, PTTH has been well characterized as the most important prothoracicotropic factor that regulates development and metamorphosis [1,2,14,24,25]. A recent study in *R. prolixus* (Hemiptera) showed that the in vitro RNAi-mediated knockdown of *PTTH* performed on brains resulted in reduced ecdysteroid production by PGs [22]. However, it is still largely unknown whether PTTH is a key neuropeptide hormone for controlling ecdysone biosynthesis and development in hemimetabolous insects. In our current study, we focused on the role of PTTH and Torso in BPH by generating PTTH-null and Torso-null mutants. Our findings uncovered the pivotal role of the PTTH–Torso signaling system in governing growth and development through regulating ecdysone biosynthesis in *N. lugens*.

In *Bombyx*, the active PTTH, synthesized and released by a duet of dorsolateral neurosecretory cell pairs within the brain, manifests as a glycosylated homodimeric neuropeptide adorned with three intricately woven intramolecular disulfide bonds, harmonized by a solitary intermolecular cysteine-cysteine bond [1,6,26,27]. Upon reaching the PGs, PTTH activates its cognate receptor Torso, a member of the receptor tyrosine kinase family, initiating the Ras–Raf–Erk signaling cascade, which is primarily required for ecdysone biosynthesis [2,14,28]. A homologous gene to *Bombyx PTTH* was identified in *N. lugens*, revealing a predicted mature peptide with 31% sequence affinity to *Bombyx* and 27% to *Drosophila* [21]. In situ HCR analysis revealed the expression of *NlPTTH* mRNA in two pairs of neurosecretory cells located in the dorsolateral region of the protocerebrum. We attempted but failed to detect *NlTorso* expression in *N. lugens* using in situ HCR, which may be due to inappropriate primers we designed for the assay.

Previous studies with isolated PGs have shown that PTTH has the potential to upregulate the transcription of ecdysteroidogenic enzymes, including *spook* (*spo*), *phantom* (*phm*), and *disembodied* (*dib*), and directly stimulate ecdysone production and release in response to PTTH [29,30,31,32,33]. In *Bombyx* and *Drosophila*, the PTTH receptor Torso is expressed predominantly in the PGs [14]. Recently, Uchibori-Asano et al. established a *PTTH* knockout line in *B. mori* and further characterization showed that the majority of *PTTH* knockout individuals exhibited severe developmental arrest in the L2 larval instar stage. Some *PTTH*-deficient larvae proceeded to smaller pupae through precocious metamorphosis, while others underwent metamorphosis into pupae of normal size after completing the normal number of larval instars [19]. However, *Torso* deficiency only caused a delay in development during the larval and pupal stages, without developmental arrest or precocious metamorphosis [20]. In this study, we generated *PTTH* and *Torso* homozygous mutant lines in *N. lugens*, respectively, employing targeted gene disruption through the CRISPR–Cas9 system. Both *PTTH*-deficient and *Torso*-deficient lines showed prolonged nymphal duration and increased body size. Similar phenotypes were observed in PTTH neuron ablation and PG-specific *Torso* silencing *Drosophila* [2,14]. In *Drosophila*, PTTH is thought to trigger the production and release of ecdysone, the precursor of the active steroid molting hormone 20E [24], resulting in elevated levels of 20E, providing a systemic signal that ends the larval growth period and initiates metamorphosis [14]. We examined 20E levels in fifth-instar nymphs at 24, 48, and 72 h post-ecdysis by ELISA, and found that 20E titers of *NlPTTH^−/−^* and *NlTorso^−/−^* mutants were significantly reduced in fifth-instar nymphs at 48 h post-ecdysis compared to *Wt* controls, in agreement with previous experiments in *Bombyx* and *Drosophila* [2,14,19,20]. Our data provide strong evidence that the PTTH–Torso signaling system is critically required to stimulate ecdysone biosynthesis and thereby controls nymphal duration and adult body size in *N. lugens*.

We performed additional analyses to investigate the effects of NlPTTH and NlTorso on the life history characteristics of BPH. Our results indicated that *NlPTTH^−/−^* and *NlTorso^−/−^* mutants displayed reduced lifespan and fecundity, as well as reduced hatching rate, compared to *Wt* controls. In *Drosophila*, MaryJane et al. demonstrated that PTTH signaling plays a pivotal role in orchestrating the harmonious integration of diverse environmental cues with the developmental program, thereby ensuring individual fitness and survival, and *PTTH* mutants displayed reduced adult survival rates under suboptimal environmental conditions, such as cases of nutritional deficiency or high population density [34]. A recent study unveiled that PTTH governs longevity by modulating immune responses in adult *Drosophila*. *PTTH* mutants exhibited prolonged lifespan in both female and male adults, constitutive knockdown of Torso PG cells extended the lifespan in female flies, and conversely, constitutive knockdown of Torso in oenocytes resulted in a shortened lifespan [35]. In *Bombyx*, Zhang et al. revealed reduced expressions of longevity pathway genes in Torso mutants [20]. Thus, the role of PTTH–Torso signaling on life history characteristics may differ in different insects, and the mechanism in BPH needs to be further explored.

In conclusion, *NlPTTH^−/−^* and *NlTorso^−/−^* mutants resulted in prolonged nymphal duration and increased adult body size through regulation of ecdysone biosynthesis, but also showed significant effects on adult lifespan, reduced female fecundity and egg hatching rates. It suggests that the PTTH–Torso signaling system is largely conserved in the regulation of developmental transition in both holometabolous and hemimetabolous insects. However, further efforts are required to unravel the mechanisms orchestrating the coupling of nutritional and environmental cues with ecdysteroidogenesis in the regulation of development in *N. lugens*.

## 4. Materials and Methods

### 4.1. Insect Rearing

The populations of BPH utilized in the current study were initially gathered in Hangzhou, China (30°16′9 N, 12°11′ E). The BPH populations were reared on rice seedlings (variety TN1) at a temperature of 26 ± 0.5 °C, with a photoperiod of 16 h light to 8 h dark and a relative humidity of 50 ± 5%.

### 4.2. Amplification and Analysis of the Sequence

Total RNA was extracted from the whole *Wt* BPH using RNAiso plus (Takara, Dalian, China) following the manufacturer’s protocol. To synthesize cDNA, 1 μg of total RNA was reverse-transcribed in a 20 μL reaction using HiScript II QRT SuperMix (Vazyme, Nanjing, China). *NlPTTH* and *NlTorso* were amplified using Green Taq Mix (Vazyme, Nanjing, China) with gene-specific primers (*NlPTTH*-F: GTCTTATGGCGCCTCATGGT, *NlPTTH*-R: AGGGTTCAATCACTTTGTCGC, *NlTorso*-F: GCGATGTGCGATGCGATGTT, *NlTorso*-R: AGCTCTCTCTGGTGTGCTGT). Prediction of the cleavage sites and the mature peptide was performed with NeuroPred, accessible at http://stagbeetle.animal.uiuc.edu/cgi-bin/neuropred.py (9 March 2024). Alignment of PTTH homologues was performed using Clustal X version 1.81 and GENEDOC version 2.5. Phylogenetic trees were constructed in MEGA 11 using the neighbor-joining method, with bootstrapping set to 1000 replicates.

### 4.3. Spatiotemporal Expression of NlPTTH and NlTorso in Wt BPHs

To investigate the temporal expression of *NlPTTH* and *NlTorso*, total RNAs were isolated from eggs in rice leaf sheaths at 0, 24, 48, 72, 96, 120, 144, and 168 h, first- to third-instar nymphs at 0, 24, and 48 h post-ecdysis, fourth-instar nymphs at 0, 24, 48, and 60 h post-ecdysis, fifth-instar nymphs at 0, 24, 48, 60, and 72 h post-ecdysis, and female adults at 0, 24, 48, 72, 96, and 120 h after eclosion. To investigate tissue-specific distribution, optic lobes, central brain, gnathal ganglia, the ventral nerve cord, salivary glands, midgut, Malpighian tubules, integument, and fat body (Fb) were dissected from fifth-instar nymphs at 0 h and ovaries and testes were dissected from female and male adults. qRT-PCR analysis was conducted utilizing a CFX96TM real-time PCR detection system (Bio-Rad, Hercules, CA, USA) in conjunction with specific primers. The 18S ribosomal RNA gene of the target organism, BPH, served as the internal control for normalization purposes. To evaluate the quantitative variation in transcript levels, the ΔΔCt method was used.

### 4.4. In Vitro Synthesis of Cas9 mRNA and Single Guide RNA (sgRNA)

The sgRNAcas9 algorithm [36] was employed to identify sgRNAs within the BPH genome, utilizing the *NlPTTH* and *NlTorso* sequences as reference. The sgRNA was transcribed in vitro employing a T7 High Yield RNA transcription kit (Vazyme, Nanjing, China) following the manufacturer’s instructions, as previously outlined [37]. *NlPTTH*-sgRNA-F (5′-T7-GGCGTAGCGCGGCCACGACTCGGTTTTAGAGCTAGAAATAGC-3′) and *NlTorso*-sgRNA-F (5′-T7-GGATCGACCACAGTAACACGCCGTTTTAGAGCTAGAAATAGC-3′) were utilized for the synthesis of sgRNA. Cas9 mRNA was transcribed in vitro from the plasmid vector, pSP6-2sNLS-SpCas9, and purified using the mMESSAGE mMACHINE SP6 Transcription Kit (Thermo Fisher Scientific, Waltham, MA, USA) and Poly(A) Tailing Kit (Invitrogen, Carlsbad, CA, USA). Embryo injection procedures were conducted following the methodology described by Xue et al. [37]. Eggs at the preblastoderm stage were carefully dissected from rice sheaths within 2 h of oviposition, and microinjection of the CRISPR–Cas9 reagent was then executed within the subsequent hour.

### 4.5. Homozygous Mutant Line Screening

To verify the mutations induced by CRISPR–Cas9, genomic DNA (gDNA) was extracted from wings and employed as a template for PCR amplification. Specific primers were used to separately span the *NlPTTH* and *NlTorso* target sites (*NlPTTH*-exon1-F:TTCGAGGTGAACCACAGCAT, *NlPTTH*-exon1-R:CGCAACACCTTGACCGGATA, *NlTorso*-exon10-F: TGCATGATCCTACCAGTTAGCC, *NlTorso*-exon10-R:CCAATCCACCAGAGGCTGTT). The PCR amplicons were validated by Sanger sequencing, and mutant G0 individuals were subsequently crossed with *Wt* BPH to generate G1 progeny. G1 nymphs were raised to adulthood and gDNA was extracted from the forewings for genotyping. G1 adults with mutations at the target site were chosen and crossed with *Wt* adults to produce G2 offspring. G2 BPHs were then crossed to generate homozygous mutants in G3.

### 4.6. In Situ Amplifiers Based on the Hybridization Chain Reaction (HCR) Mechanism

A multiplexed HCR v3.0 protocol was implemented for the detection of *NlPTTH* expression. Hybridization buffer, HCR probes, wash solution, amplification buffer, and fluorescently labeled hairpins were purchased commercially (Molecular Instruments Inc., Los Angeles, CA, USA). The immunofluorescence assay was performed following the manufacturer’s instructions. Briefly, samples were prepared and fixed in 4% paraformaldehyde (PFA) overnight at 4 °C. Following fixation, samples underwent five washes with 0.1% PBST (0.1% Tween 20 in PBS), then were treated with 70% ethanol and rehydrated with a gradient of PBST in methanol (25%, 50%, 75%) and five times at 100%. Subsequently, samples were postfixed with 4% PFA for 25 min at room temperature and subsequently subjected to five washes with PBST. Then came the detection stage: samples were incubated in probe solution (2 pmol of each probe set) overnight (12–16 h) at 37 °C after pre-hybridization in probe hybridization buffer for 30 min. On the subsequent day, the samples underwent three washes with 5× SSCT at room temperature before proceeding to the amplification stage. Samples were first pre-amplified in amplification buffer for 30 min at room temperature. They were then incubated in hairpin solution, consisting of 30 pmol of hairpins h1 and h2 added to the amplification buffer after heating at 95 °C for 90 s, followed by cooling to room temperature in a dark drawer for 30 min. This incubation was maintained at room temperature overnight (12–16 h). On the third day, excess hairpins were washed several times with 5× SSCT at room temperature. Following the completion of these procedures, the samples underwent mounting in anti-fade mounting medium and visualization by fluorescence microscopy utilizing a Zeiss LSM 880 confocal microscope (Carl Zeiss MicroImaging, Göttingen, Germany).

### 4.7. Determination of 20-Hydroxyecdysone (20E)

Fifth-instar nymphs were collected at 24, 48, and 72 h post-ecdysis from *Wt*, *NlPTTH*, and *NlTorso* mutants. Ecdysteroids were subsequently extracted from the entire bodies of *N. lugens* according to Nakaoka et al. with some modifications [38]. Nymphs were homogenized and sonicated for 20 min in 200 μL sonicated buffer containing 50 mM Tris-HCl, pH 7.5, 150 mM NaCl and 2 mM EGTA. A quantity of 400 μL of 1-butanol was added to the samples to extract ecdysteroids by vortexing for 5 min and centrifugation at 1000× *g* for 10 min. For complete extraction of ecdysteroids from the nymphs, three repetitions of the 1-butanol treatment were performed. The supernatants were collected and subsequently evaporated in a centrifugal evaporator (Eppendorf, Hamburg, Germany). ELISA was performed using a commercially available Insect 20-Hydroxyecdysone Enzyme Immunoassay Kit (Arbor Assays, Ann Arbor, MI, USA) according to the manufacturer’s instructions.

### 4.8. Body Weight, Duration of Embryonic and Nymphal Stages and Adult Lifespan

For the assessment of BPH body weight, fourth-instar nymphs from *Wt*, *NlPTTH^−^^/−^*, and *NlTorso^−^^/−^* strains were gathered and allowed to develop to the fifth-instar stage. Fifth-instar nymphs were collected at 24, 48, and 72 h post-ecdysis and pooled (n = 8) for weighing. For the determination of embryonic stage duration, a cohort comprising 100 female insects was introduced onto fresh rice seedlings to facilitate oviposition, which occurred over a period of 2 h. Newly emerged nymphs were observed and recorded. To assess the duration of the nymphal stage, first-instar nymphs were collected immediately after hatching and reared to adulthood. Emerging adults were observed and recorded. To evaluate lifespan, female and male adults were collected within 0–2 h after eclosion (hAE) and placed on fresh rice seedlings for rearing. The survival of each adult was assessed and recorded every 12 h.

### 4.9. Fecundity and Hatching Rate Analysis

Female and male adults were collected for a paired mating assay within 0–12 hAE for fecundity analysis. Each female adult was paired with two male adults and provided the opportunity to lay eggs over a span of 10 days. Subsequently, the eggs were counted following the removal of the adult insects. To calculate the hatching rate, a group of 100 females was introduced to fresh rice seedlings to deposit eggs for 2 h. Following this, both the hatched nymphs and unhatched eggs were counted.

### 4.10. Statistical Analysis

Statistical analyses were performed with GraphPad Prism (v. 9.5.0). Results were analyzed using one-way ANOVA test, two-tailed Student’s *t*-test, two-way ANOVA and log-rank (Mantel–Cox) test. Data are presented as means ± standard error of the mean (SEM) from independent biological replicates. Different lowercase letters mean significant differences (*p* < 0.05); ns indicates no significant difference between two groups; * indicates significant difference between two groups at *p* < 0.05; ** indicates significant difference between two groups at *p* < 0.01; *** indicates significant difference between two groups at *p* < 0.001; and **** indicates significant difference between two groups at *p* < 0.0001.

## Data Availability

The homozygous mutant lines are available from the authors upon request.
